# Influences of wind speed and direction on atmospheric particle concentrations and industrially induced noise

**DOI:** 10.1186/s40064-016-3553-y

**Published:** 2016-10-28

**Authors:** Ayansina Ayanlade, Ebunoluwa Folasade Oyegbade

**Affiliations:** Department of Geography, Obafemi Awolowo University, Ile-Ife, Nigeria

**Keywords:** Wind speed/direction, Atmospheric particles, Turbulent, Noise levels

## Abstract

**Purpose:**

In this study, the spatial and temporal relationship of wind speed, atmospheric particles concentration, and the industrial-induced noise levels during different times of the day were examined, using sawmill industrial location around Ile-Ife in Osun of Nigeria as a case study.

**Methods:**

Mobile devices were used to measure noise level and basic meteorological parameters were examined and their influences on the noise levels distribution were assessed. The maximum and minimum sound levels; L_max_ and L_min_, the PM_10_ and PM_1_ particle concentrations, wind speeds and directions were measured in the morning (7–9 a.m.), afternoon (12–2) and evening (4–6 p.m.) over 14 consecutive days.

**Results:**

The results revealed that the noise level varies spatiotemporally, much more consistent spatial distribution along the vicinity of sawmill industries. A higher level of noise occurred during the weekday (WD), L_eq_ > 70 dB(A), compared to weekends (WE). Extreme average noise levels are associated with the immediate neighbourhood of sawmill industrial areas during WD compared to streets and road annexes of the study area. The results also show a very weak relationship between noise and PM_10_ and PM_coarse_ for both WD and WE with r < 0.35 for PM_1_ and r < 0.20 for PM_coarse._ There appears to be a moderate significant correlation between noise level and PM_1_ during some meteorological conditions with r > 0.51.

**Conclusion:**

The slight relationship between noise and PM_1_ is perhaps a result of wind movement that carries particles from the source region since booth noise and particles mostly originate from the sawmill. The study concludes that wind speeds and directions have a significant influence on both noise level and particle concentration within the study sites.

## Background

Noise pollution has been one of the environmental hazards as early as the inception of civilization to the recent era of technology. Noise pollution has been associated with human activities and persistent human interaction with the environment. Over the past decades, noise pollution has received increased attention and studies have reported that noise pollution is one of the environmental hazards affecting human, the effects range from annoyance to difficulty in falling asleep, which latter leads to high blood pressure (WHO 2005; Ugwuanyi et al. 2005; Den Boer and Schroten 2007; Tetreault et al. 2013). Studies have stated that noise pollution causes hearing impairment, physiological and mental illness, and in many cases prompts behavioural and social effects (Den Boer and Schroten 2007; Pathak et al. 2008; Weber 2009; Ballesteros et al. 2010; WHO 2011, ​^2013^; Lee 2014). A study by De Vos and Van Beek (2011) has revealed that about two billion people in cities around the world are subject to over 55 dB(A) noise level. But, a report by the European Environment Agency, EEA (2014) estimated that nearly 115 million people in Europe are exposed to average day/night time noise levels of about 55 dB(A). The findings from these studies revealed that noise pollution is severe in urban areas, especially in less developed countries where insufficient control is exercised, mainly if the cities are poorly planned (Pathak et al. 2008; Foraster 2013).

The need to understand environmental noise and its impacts on people in urban areas has driven other researchers in both developed and developing countries. In developed countries, several noise pollution guidelines have been developed. In sub-Saharan Africa, although, where noise legislations exist, they are often poorly enforced and implemented (Sonibare et al. 2004; Chung et al. 2005; Oyedepo and Saadu 2009; Sørensen et al. 2011; Oyedepo 2012). However, interest may be attributed to the current development of guidelines and standards for environmental pollution by the Federal Environmental Protection Agency (FEPA 1991). Unlike cities in developed countries, surprisingly, few studies have been carried out in sub-Saharan Africa which assessed spatial and temporal patterns in noise level and relate this to meteorological parameters. There is a need for examination of atmospheric air pollution and environmental noise, their relationship and how this could affect cities inhabitants. Thus, this study aims at evaluating the spatial and temporal variations in the noise levels during different times of the day and examines influences of the wind on industrial-induced noise pollution in sawmills industrial location around Ile-Ife in Osun of Nigeria. The study focuses on assessing the spatiotemporal relationship between wind speed and direction and noise level and determine the extent noise and particles have a common cause. The basic meteorological parameters were examined and their influences on the distribution of industrial-induced and traffic noise were assessed.

## Methods

**Fig. 1:**
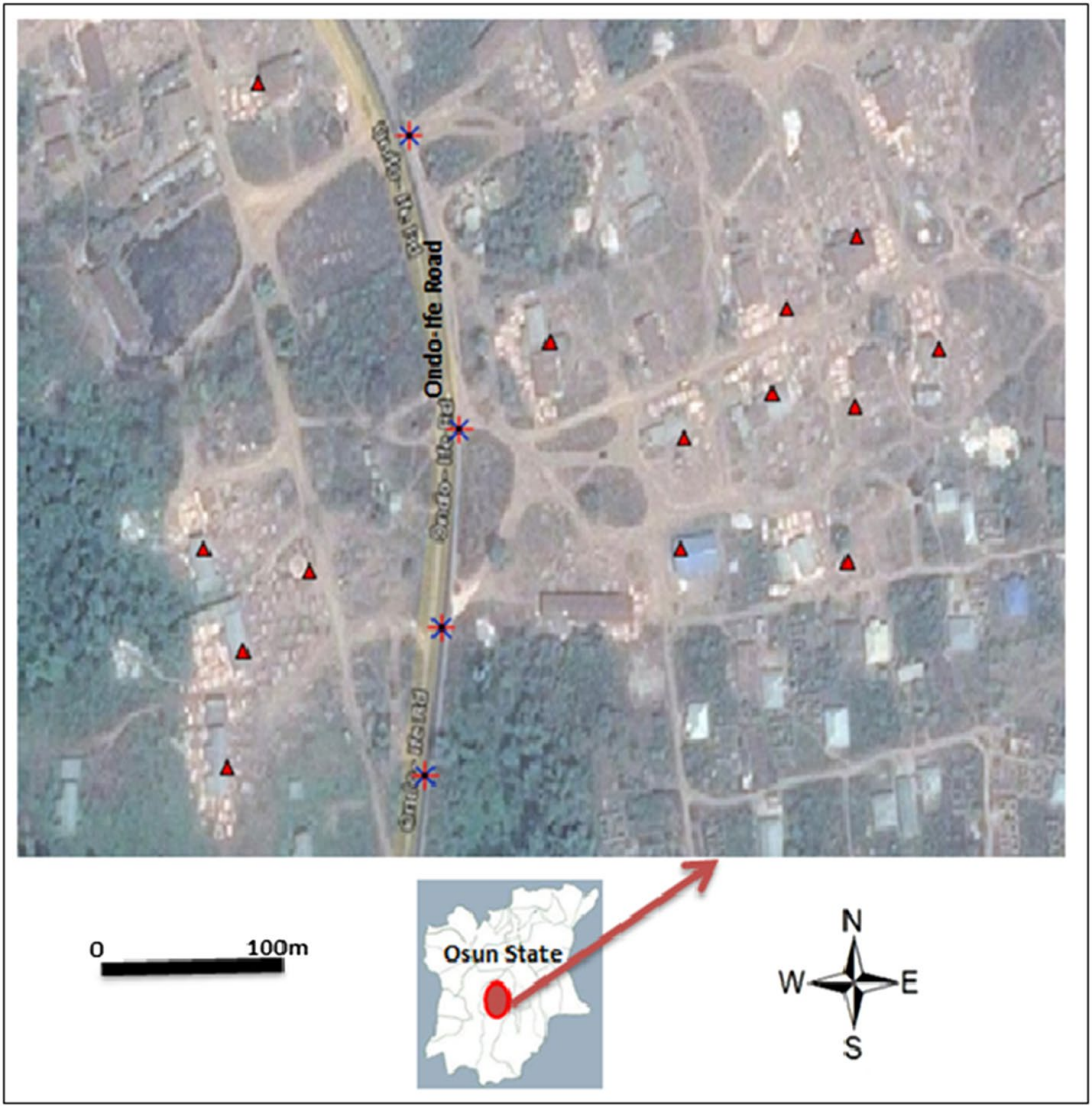
Maps of the study site. Map of Osun state in *green*. *Triangles* represent locations of sawmill industrial engines while *star* symbols represent locations of measurements along the roadsides. Modified from Google earth

**Fig. 2:**
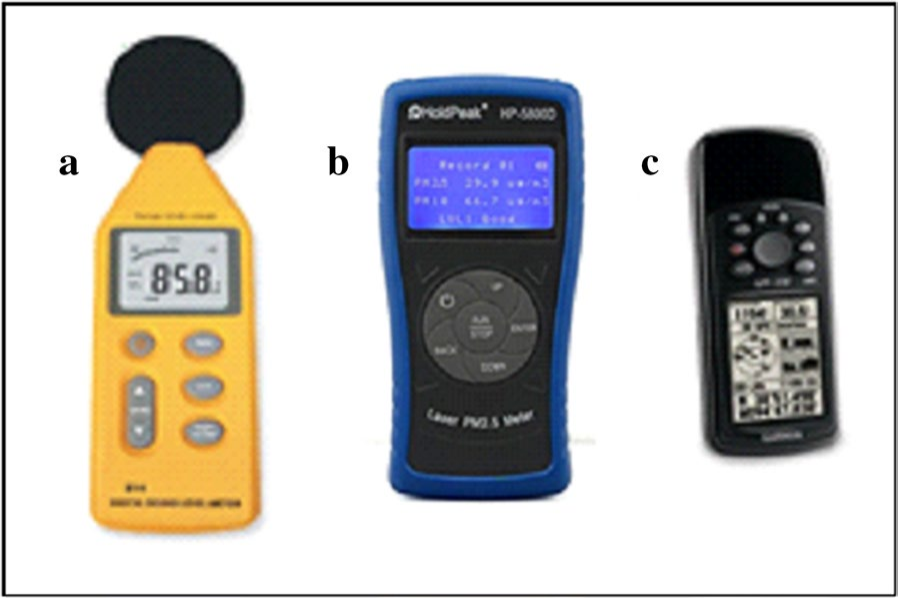
Mobile equipment used to measured noise level (**a**), particle concentrations (**b**) and sample locations (**c**)

The sites are within Ita-Osa and Erefe communities of Ile-Ife in Osun, Nigeria (Fig. 1). Climate regime in this area is tropical humid climate. The mean annual rainfall is bimodal in nature with peaks in July (average of 350 mm) and September (average of 200 mm). The mean minimum temperature ranges from 20 °C in January to 23 °C in February, while the mean maximum for the hottest month is 28.6 °C (Adejuwon and Jeje 1975; Ojo 1977). One of the major activities within the communities is the enormous sawmills industries, due to the proximate of the area to tropical forest exploitation and lumbering. Samples were chosen from Ita-Osa and Erefe, out of many communities in IELG because the communities are the major location where sawmills industrial activities are been carried out in IELG. Noise level and atmospheric particles concentration were measured simultaneously, using mobile sensors. The measurements were taken along sawmill industrial locations, at some road junctions, and some residential areas for approximately 45 min at a time. A set of digital mobile noise meters (SET 1350) was used to measure noise level at ten repeated points (Fig. 2a). The types of sound level meter used are the typical mobile type which consists of a microphone for picking up the sound and convert into an electrical signal. The device is calibrated to read the sound level in dB(A). The noise meter had a measuring level range of 35–130 dB(A) with Accuracy up to ±1.5 dB. In every measuring location, the microphones were pointed in the general direction of the noise source at a distance, not less than 1 m away from any reflecting objects. At the start of measurement, noise levels were measured first at the position of the machines at sawmill and other measurements were taken at intervals of 30 feet outward from each sawmill, in all directions, in the morning (7–9 a.m.), afternoon (12–2) and evening (4–6 p.m.) during 14 consecutive days (from Monday to Sunday). The maximum and minimum sound levels (L_max_ and L_min_), were measured, the highest and the lowest time-weighted sound levels were also measured in dB(A) while the equivalent sound pressure level L_eq_ were also calculated for different time slices.

Revisiting the sample locations was guided by Global Positioning System (GPS). Sets of Garmin GPSs with 0.2% resolution accuracy (Fig. 2c) were used in the field to locate sample points and record the coordinates of all sample points for effective mapping. Noise levels for both weekdays (WD) and weekends (WE) were mapped using inverse distance weighting (IDW) interpolation technique as stated by Burrough and Rachael 1998; Ayanlade 2009; Samanta et al. 2012; Pokhrel et al. 2013. This was performing in Quantum GIS environment. IDW was calculated using weights change which was based on the linear distance of the noise sample points from the unsampled points in inverse proportions. The implication of this on noise measurement is that noise level values closer to the un-sampled locations are more representative of the value to be estimated than values from samples farther away. Thus, IDW was used to interpolate deviations from a long-term mean in noise level data over the study periods.

The measurement of wind speed, direction and turbulence were carried out using set mobile anemometers which were installed on poles at a height of 3.75 m, with a 1 min resolution. This implies that the wind data were gathered above rooftop height and within the street. The PM_10_ and PM_1_ concentrations were measured using mobile optical particle counters. A Set of HP 5800D PM2.5/PM10/Formaldehyde Monitor Detector model with resolution: ±0.1 μgm^−3^ were used in this study (Fig. 2b). Studies have shown that mobile optical particle measurement method is a useful method for assessing spatial and temporal variability in the air pollutant concentrations (Tang and Wang 2006, 2007; Kaur et al. 2007; Weber and Litschke 2008; Weber 2009). In the present study, differences between spatial distribution of fine (PM_1_) and coarse particle (PM_coarse_) size fraction were examined while the spatial average of particle concentrations was calculated for coarse particle size fraction using methods as in Weber and Litschke (2008):

1 $$PM_{coarse} = PM_{10} - PM_{1}$$

where *PM*_10_ and *PM*_1_ represent the particulate material with aerodynamic diameters ≤10 µm and ≤1 µm respectively; while *PM*_*coarse*_ is the differences between the particulate matters of aerodynamic diameter between 10 and 1 μm. Descriptive statistical techniques were used to find the average noise level and average particle concentrations for both weekdays and weekends. Correlation between particles concentration and noise were calculated to examine the spatiotemporal relationships between particles concentration, wind speed and noise levels. Correlation between particles concentration and noise were calculated in terms of Pearson’s correlation coefficient “r”, the magnitude of the correlation coefficients varies between ±1. The spatial correlation between noise and particles, under conditions of turbulence mixing, was calculated for all measurement sites and their covariance was also assessed based on meteorological conditions and time of day when noise levels were measured.

## Result and discussion

### Noise level at different sampling sites

**Fig. 3:**
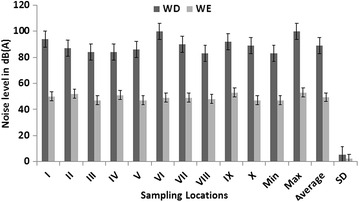
Measurement of L_eq_, at different sampling sites during 7–9 a.m. WE symbolizes weekend while WD symbolizes for the weekdays

**Fig. 4:**
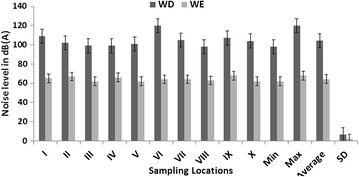
Measurement of L_eq_, at different sampling sites during 12–2 p.m. WE symbolizes weekend while WD symbolizes for weekdays

**Fig. 5:**
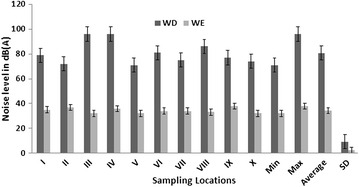
Measurement of L_eq_, at different sampling sites during 4–6 p.m. WE symbolizes weekend while WD symbolizes for weekdays

The noise level varied spatially and temporally over the periods of study, as detailed in Figs. 3, 4 and 5. These figures present variations in noise level, min, max and standard deviation for both WE and WE. Two major patterns are noticeable from these results: (1) the noise levels at different sample sites vary per time, much more during 12–2 p.m., with the standard deviation is greatest between 4 and 6 p.m.; and (2) the levels of noise were higher during the WD then WE periods. The highest level of noise was recorded during the afternoon between the hours of 12 noon and 2 p.m. with L_eq_ range of 98–120 dB(A) (Fig. 4). On the other hand, the least noise level was recorded during the evening; L_eq_ range of 71–96 dB(A), within the hours of 4–6 p.m. in all measuring sites (Figs. 3, 4). The noise level during the early hours of the days between 7 and 9 a.m. with L_eq_ range of 83–98 dB(A), were higher than that of evening periods in all study sites, but much more in measuring sites IV and VI (Figs. 3, 5). The reasons for these patterns of noise levels in the study area are obvious. During the early hour of the day, the majority of sawmills industries are not usually functioning but most of the sawmill production are frequent during the afternoon periods. Observations during field work show that most of the sawmill machines were operational during the afternoon periods. The least noise level was recorded during the evening time (4–6 p.m.) because this period is the closing hours for many of sawmill industries, but much of the noise level recorded during this period were the effects of vehicles movements on nearby highway roads.

**Fig. 6:**
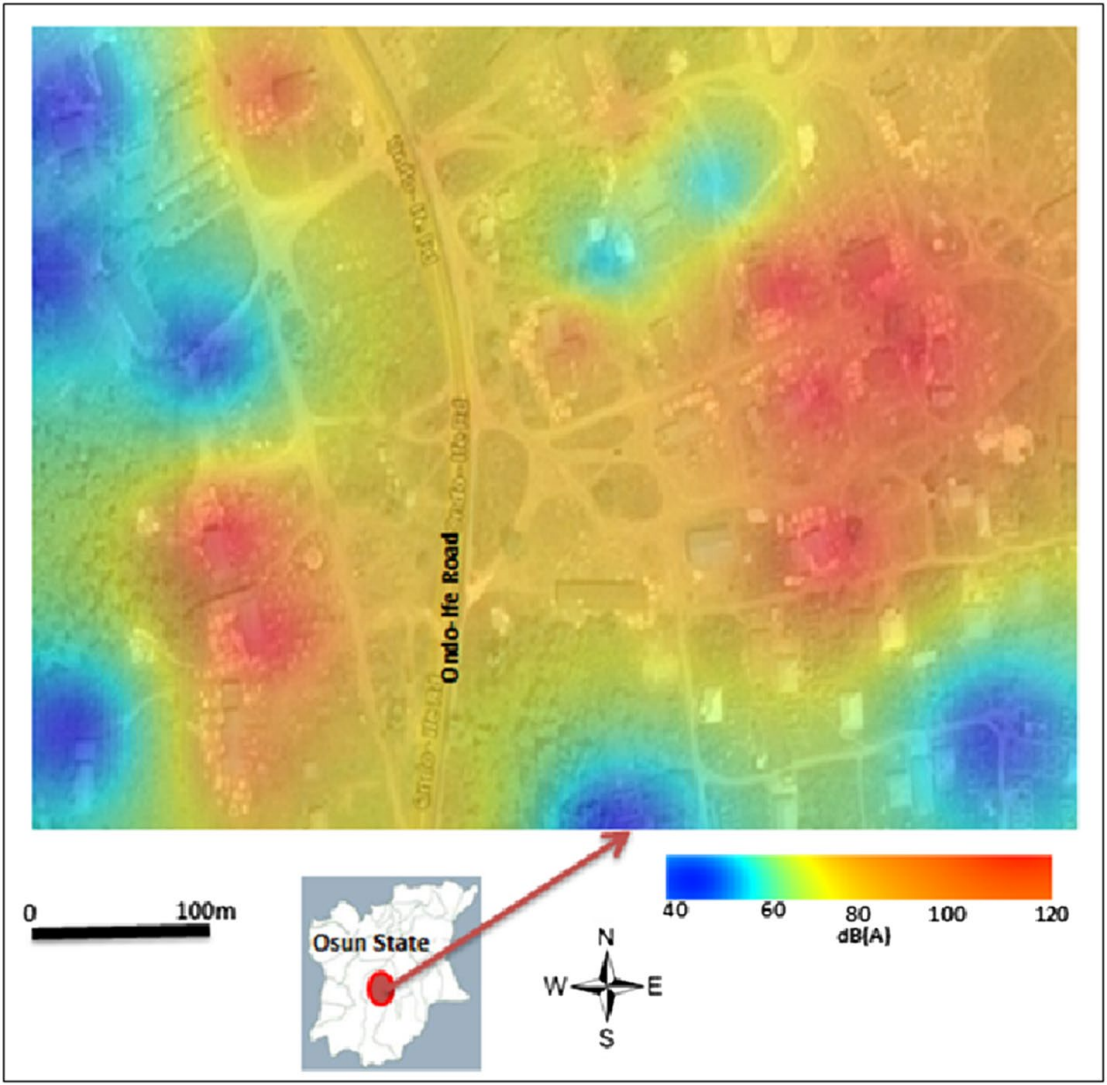
Average spatial distribution of weekdays noise L_eq_ over the period of two week’s from 25th January to 9th of February 2015. Map of Osun state in *green*

The results reveal not only daily variations in noise level but also variation between WD and WE. In general, a higher level of noise was observed during WD compared to WE (Figs. 3, 4, 5). In all study sites, the noise level L_eq_ > 50 dB(A) was recorded during WD in all time episodes, especially at the measuring sites closer to sawmill engines (Fig. 6). Conversely, noise level during WE was slightly lower, with L_eq_ range of 32–53 dB(A) (Fig. 6), but considerable small levels of noise were noted during the afternoon periods on some weekends with L_eq_ range of 55–65 dB(A). This is because some sawmill industries working during the afternoon in WE while the evening of WE is relatively the less noisy periods of time in the study area.

**Fig. 7:**
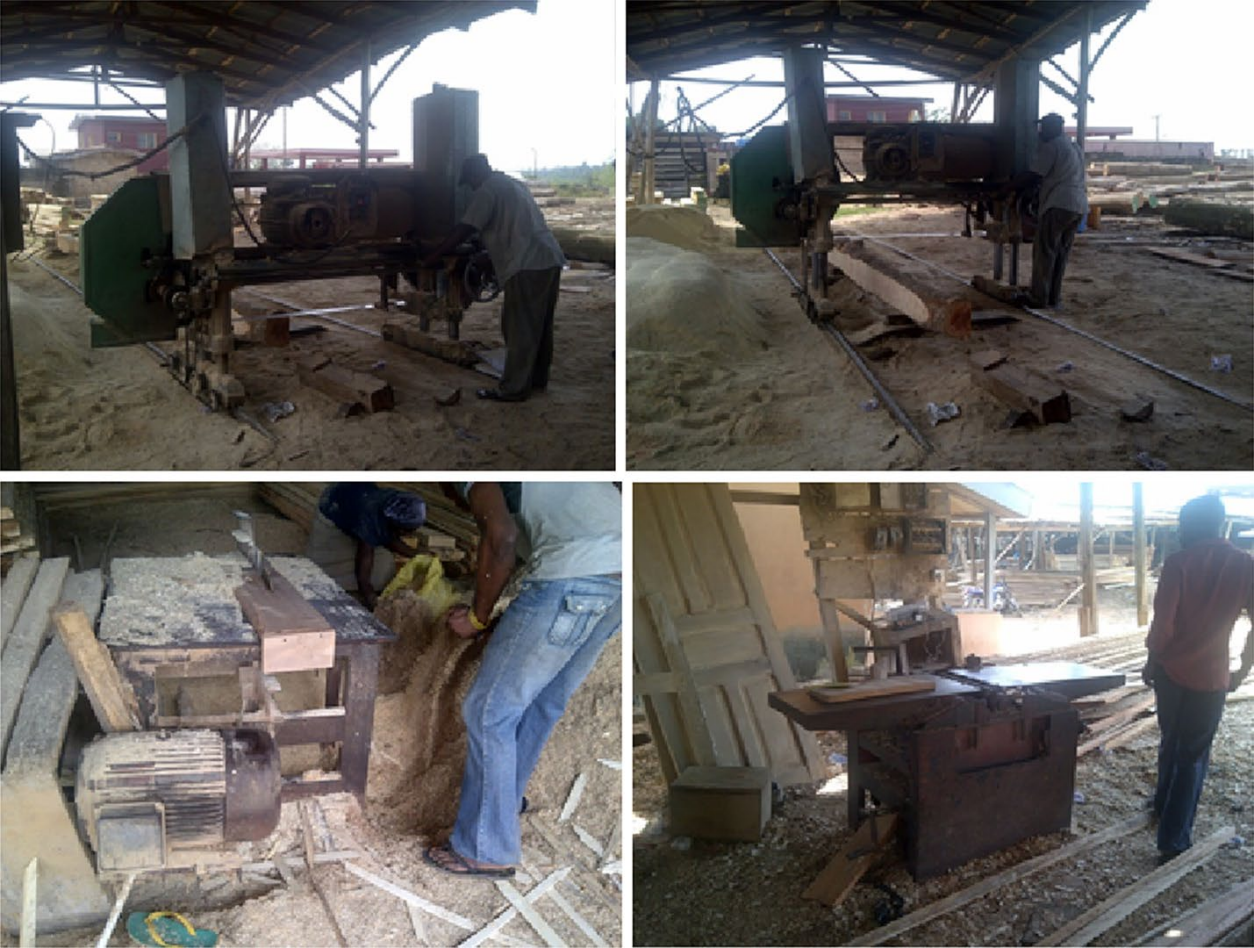
Some sawmilling activities observed during the study. Source: photo taken during the field work

Besides, the noise level also varies spatially. The highest noise level was observed in the eastern part of the study area (Fig. 6). In this location, the noise level recorded in the majority of measuring sites were L_eq_ > 80 dB(A), especially during WD. The reason for this might be because of the presence of several sawmills machines in the eastern part of the study area compared to other parts. Another reason for spatial variations in the noise level during the WD and afternoon periods is because different types of sawmill engines are being used around the study area (Fig. 7). The rate of noise pollution from these engines varies by their sizes, thus results in variation in spatial distribution of the noise, but much more in the eastern part of the study area. An unfortunate thing is the presence of some residential buildings in the eastern part of the study area. Though the present study does not investigate the societal and health implications of the high level of noise in this location, this will form part of the future study of this research. The general observation is that both weekdays and weekends noise levels exceed the noise limits standard by EPA for industrial noise levels.

**Fig. 8:**
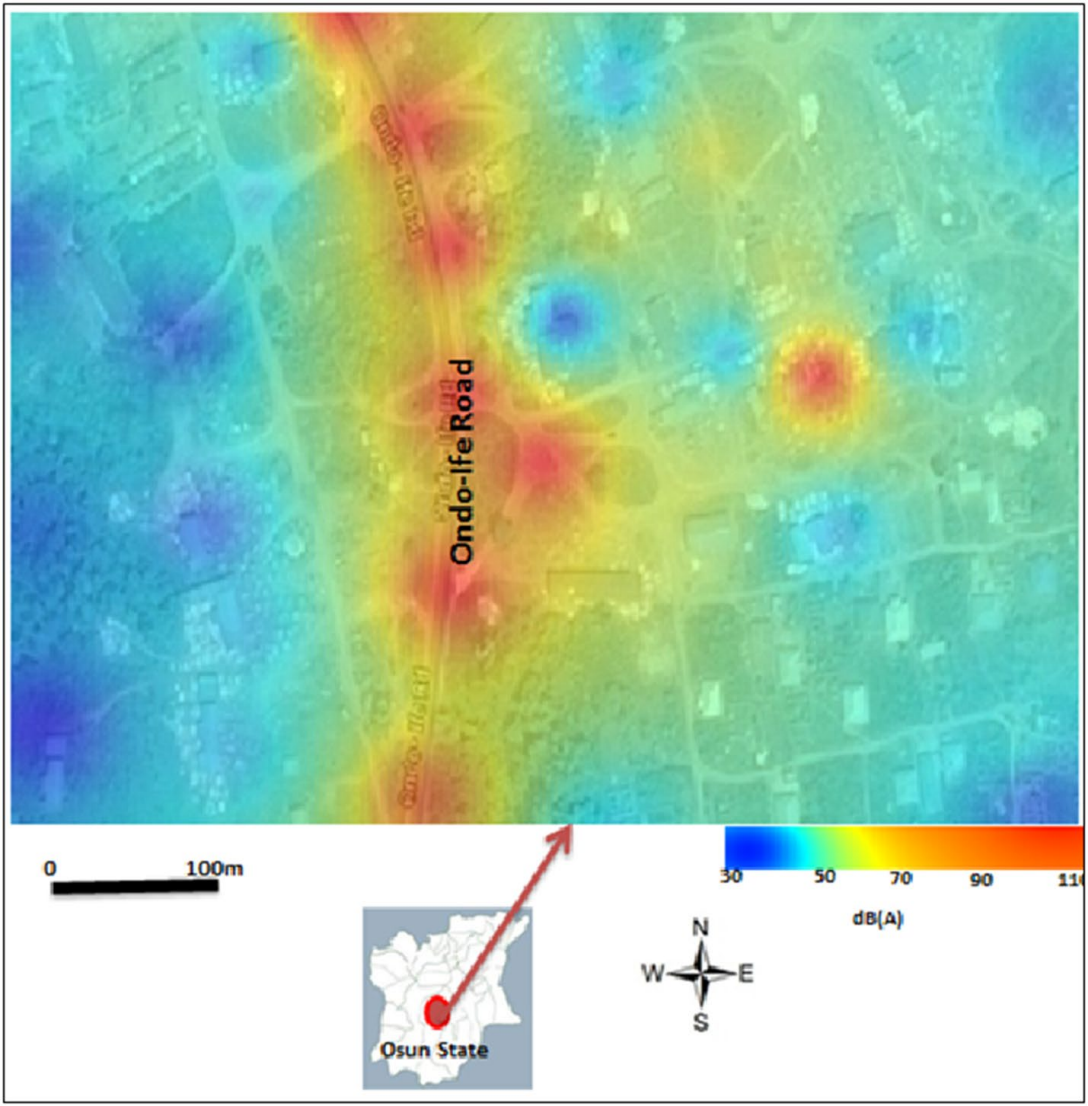
Average spatial distribution of weekends noise L_eq_ over the period of two week’s from 25th January to 9th of February 2015. Map of Osun state in *green*

What is obvious from this results is that the extreme average noise level of about L_eq_ 97–120 dB(A) are associated with the immediate neighbourhood of sawmill industrial areas during WD, while noise level of about L_eq_ 60–80 dB(A) are recorded along streets and roadsides (Fig. 6). Contrary results were obtained during the WE, when the major source of noise was characterised by the roadsides (Fig. 8). These results imply that sawmill industries are the major sources of noise in the study area during the WD, but much of noise during the WE come from road traffics, especially along the highway of Ondo-Ife Rd (see Figs. 6, 8). Generally, noise levels were somewhat lower on the street compared to the highway during WE (Can et al. 2011).

### Meteorological and pollutant situation during the measurement periods

**Fig. 9:**
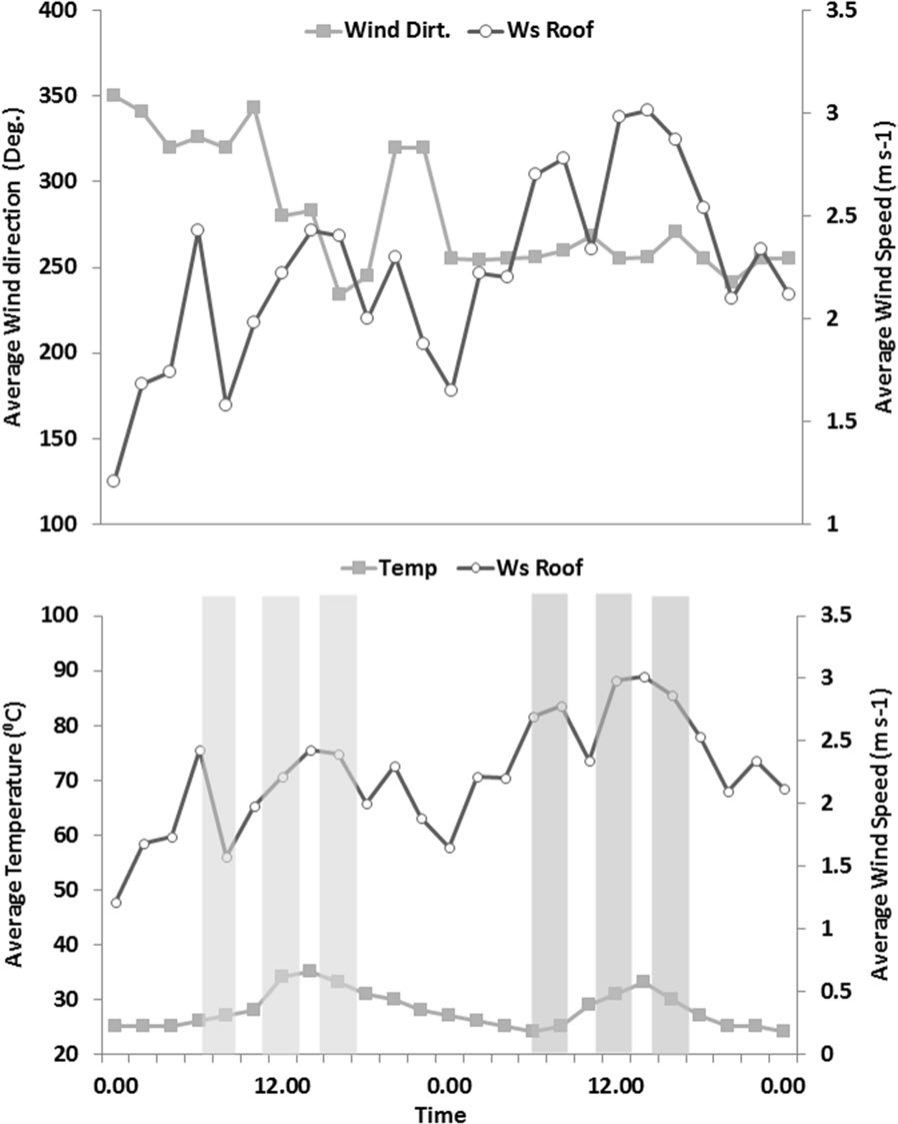
Time series of different meteorological measurement over two week’s from 25th January to 9th of February 2015. The *grey shaded bars* indicate time periods of noise and particle concentration measurements. Temp, Ws Roof and Wind Dirt stand for temperature wind speed and direction measurements, *light gray* indicate the week 1 while *dark gray* indicates the week 2

Daily average temperature and wind speeds were characterised by distinct meteorological conditions. Figure 9 illustrates the time series of average daily temperature, wind speeds and direction over study periods when noise level and particle concentration measurements were taken. Table 1 presents daily averages of different meteorological quantities during week 1 and 2, with standard deviation (SD) and maximum (Max) values. Both weeks were characterized by different weed speeds condition. Largely, week 1 was characterized by weaker wind speeds, but higher average wind speeds, mainly from the Southwest direction, occurred during week 2 (Table 1; Fig. 9). The average wind direction was 302° at ±36.04 SD and 256° at ±7.15 SD for weeks 1 and 2 respectively. No precipitation occurred during the measuring periods throughout the weeks (Table 1). There appears to be relatively uniform average temperature for both weeks, but a distinct diurnal variation in temperature intensity was recorded. The midnight and early hours of the day were characterized by lower temperatures, ranging from 24 to 27 °C while noon and afternoon periods were characterized by high temperature, ranging from 34 to 35 °C (Fig. 9). During the measuring periods, the particle concentration varied temporally in all study sites with average larger for PM_coarse_ and smaller for PM_1_ (Table 1). The particle concentration patterns are influenced by meteorology conditions. Greater average particle concentrations occurred during week 1 compared to week 2. This might be as a result of higher background aerosol from the sawmill industries and a higher percentage of relatively low wind speed which is more during measuring periods in week 1 compared to week 2 (Table 1).

**Table 1:** Daily averages of different meteorological measures during both weeks

Measurement	Week 1	Week 2
Noise level L_eq_ (dB[A])	95.95 ± *15.51* (112)	96.84 ± *14.48* (120)
PM_1_ (µg m^−3^)	32.21 ± *12.82* (53)	28.18 ± *9.73* (45)
PM_10_ (µg m^−3^)	49.32 ± *18.31* (73)	33.41 ± *13.01* (58)
Ws (m s^−1^)	1.55 ± *0.82* (2.42)	2.20 ± *0.55* (3.01)
Wind direction (deg.)	302 ± *36.04* (350)	256 ± *7.15* (268)
Rainfall (mm)	0.0	0.0
Temperature (°C)	29.71 ± *1.41* (39)	27.23 ± *2.86* (33)

Standard deviation in ​italic values, maximum values in brackets

*Ws* wind speed

### Correlation analysis of noise and meteorological situation during the measurement periods

Finally, the relationship between noise levels and meteorology parameters were examined. The correlations results show a general weak relationship. The sound levels are weakly correlated to particle concentrations, perhaps because they have the common source—sawmill. For example, PM_coarse_ were weakly associated with noise levels in both week 1 and week 2 with correlation coefficients between 0.14 < r > 0.34 (Table 2), and for WD and WE, with correlation coefficients between −0.16 < r > −0.14 (Table 3). There are to be a slight relationship between noise level and PM_1_ during morning and afternoon periods of week 1 with r = 0.51 and 0.58 respectively (Table 2). The slight correlation relationship between noise and PM_1_ during week 1 is thought to be as a result of weak in wind and change in wind direction (Table 1). Generally, noise levels demonstrate a very consistent spatial distribution along the vicinity of sawmill industries and this is independent of meteorology conditions, though, the change in wind direction and weak turbulent mixing, probably, resulted in the limited dispersion of particle during measurement in week 1. This fact is evident when comparing the correlation relation between noise and meteorology conditions during the WD and WE as detailed in Table 3. There appear a very weak relationship between noise and particle concentrations for both WD and WE for PM_1_ (r < 0.35 for WD and r < 0.25 for WE) and PM_coarse_ (r < 0.20 for WD and r < 0.14 for WE). The relationship was negative between L_eq_ and PM_coarse_ during the WE (Table 3), but moderate significant correlation relationship was obtained for L_eq_ and the number of working sawmilling machines within each study site. The results show that the higher the number of sawmilling machines the more the noise especially during the WD with r = 0.62 (Table 3), though change in wind direction and turbulent mixing affects both the noise level and particle concentrations.

**Table 2:** Correlations between noise level and particle concentrations during the two weeks of measurement

Time	Ws vs PM_1_	L_eq_ vs PM_1_	L_eq_ vs PM_coarse_
W1			
Morning	0.48	0.51	0.20*
Afternoon	0.49	0.58	0.34
Evening	−0.19*	0.21*	−0.12*
W2			
Morning	0.22	0.28	0.19*
Afternoon	0.21	0.39	0.34
Evening	−0.14	0.22	0.14*

* Not significant at p < 0.05. W1 and W2 stand for week 1 and 2 respectively. Ws represents wind speed

**Table 3:** Correlations between noise level, number of working sawmilling machines and particle concentrations during measurement (comparing WD and WE)

Measurment	Time	WD	WE
L_eq_ vs no of working sawmilling machines	Morning	0.24	0.21
	Afternoon	0.62*	0.29
	Evening	0.19	0.03
L_eq_ vs PM_1_	Morning	0.31	0.20
	Afternoon	0.32	0.24
	Evening	0.34	0.14
L_eq_ vs PM_coarse_	Morning	−0.17	−0.14
	Afternoon	0.20	−0.18
	Evening	−0.18	−0.16

* Significant at p < 0.05. WD and WE represent weekdays and weekends respectively

## Conclusion

This study draws upon two set of data; noise measurement and meteorological data to examine spatial and temporal variation in wind and it influences on the noise level. The samples measurements were conducted during the period from 26 January through 9 February 2015 in sawmill industrial area in Ile-Ife. Using mobile measurements, the study aims at assessing effects of wind speed/direction on industrial-induced noise. The results from this study revealed that: (1) noise levels in the study area are by far exceeding the EPA noise standard for industrial area; (2) noise level demonstrate a very consistent spatial distribution, higher along the vicinity of sawmill industries and this is independent of meteorology conditions; (3) there are slight correlation relationship between noise and PM_1_ due to weak turbulent mixing, during week 1, resulted from limited dispersion of particle during measurement; (4) extreme average noise levels are associated with the immediate neighbourhood of sawmill industrial areas during WD compared to streets and road annexes of the study area. However, noise levels were more along the highway roadside during WE when the majority of the sawmill industries were not in operation.

The results from this study revealed that, in many cases, particle sizes do not have a similar response to meteorological conditions and that wind speeds and directions have significantly influence on both noise level and particle concentration (Jamriska and Morawska 2008; Nicolas et al. 2009; Can et al. 2011). These results imply that particle concentrations and distributions are strongly varied with wind speed and direction as they move away from the major source (Zhang et al. 2004; Muzet 2007; Owoade et al. 2013). The influences of meteorological conditions on noise levels appears much more complex. The results show that the correlation between noise level and PM1 appears relatively moderate during weak turbulent metrological conditions. Previous studies have earlier reported a moderate correlation between noise level and atmospheric particles. The foremost studies include Weber and Litschke (2008) and Davies et al. (2009) which established a slight relationship between some particle concentrations and noise levels. The major finding of the present study is that noise levels and atmospheric particles sensitive to wind speeds and directions as they cover distances from the sources. It is also obvious from this study that sawmill mechanical noise is the major sources of the noise in the study area, noise that results from all kinds of sawmill operation and power capacity engines. Generally, noise levels demonstrate very consistent spatial distributions along locality of sawmill industries and this is independent of meteorology conditions. But, weak turbulent mixing results to moderate association between noise level and some particle concentrations. The spatial correlation between particle concentrations and the noise level was largely weak as a result of higher turbulent mixing and some changes in the direction of the ambient wind during measurement periods.
